# Survival prognosis model for elderly women with epithelial ovarian cancer based on the SEER database

**DOI:** 10.3389/fonc.2023.1257615

**Published:** 2023-09-29

**Authors:** Yingping Zhou, Aifen Wang, Xin Sun, Rong Zhang, Luwen Zhao

**Affiliations:** ^1^ The First Department of Gynecology, Affiliated Hospital of Chengde Medical University, Chengde, Hebei, China; ^2^ The First Department of General Surgery, Affiliated Hospital of Chengde Medical University, Chengde, Hebei, China

**Keywords:** elderly women, epithelial ovarian cancer, SEER database, prognostic factors, nomogram

## Abstract

**Objectives:**

We aimed to analyze the risk factors of elderly women with epithelial ovarian cancer (EOC) using data on the SEER database, and to generate a nomogram model their 1-, 3-, and 5-year prognoses. The resulting nomogram model should be useful for clinical diagnoses and treatment.

**Methods:**

We collected clinical data of women older than 70 years with epithelial ovarian cancer (diagnosed on the basis of surgical pathology) from the SEER database including datasets between 2010 and 2019. We randomly grouped the data into two groups (7:3 ratio) using the R language software. We divided the independent prognostic factors obtained by univariate and multi-factor Cox regression analyses into training and validation sets, and we plotted the same independent prognostic factors in a nomogram model of overall survival (OS) at 1, 3, and 5 years. We used the C-index, calibration curve, and area under the curve to validate the nomograms. We further evaluated the model and its clinical applicability using decision curve analyses.

**Results:**

We identified age, race, marital status, histological type, AJCC staging, differentiation degree, unilateral and bilateral tumor involvement, number of positive lymph nodes, chemotherapy, surgery, sequence of systemic treatment versus surgery, and time from diagnosis to treatment as independent prognostic factors for elderly women with EOC (*P* < 0.5). The C-indexes were 0.749 and 0.735 in the training and validation sets, respectively; the ROC curves showed that the AUC of each prognostic factor was greater than 0.7; and, the AUC values predicted by the line plot were similar in the training and validation sets. The decision curves suggest that this line plot model has a high clinical value for predicting overall survivals at 1, 3, and 5 years in elderly women with EOC.

**Conclusion:**

The nomogram model in this study can provide an accurate assessment of the overall survival of women older than 70 years with EOC at the time of the first treatment, and it provides a basis for individualized clinical treatment.

## 1 Introduction

Ovarian cancer is the leading cause of death among the gynecologic cancers (1) and the fifth most common cause of death after lung, breast, colorectal, and pancreatic cancers (2). In addition, ovarian cancer is the most difficult gynecologic malignancy to treat in the clinical practice. Ovarian cancer occurs mostly in postmenopausal women. The main organs of the human body undergo gradual functional decline with age, and as a result elderly patients eshibit relatively poor tolerance to malignant tumors. The mortality rate of ovarian cancer has not declined satisfactorily since 1980 (3), but its incidence has shown a yearly decline in countries, such as the United Kingdom, France, Germany, and the United States (4). Accurate prognoses for elderly women with EOC would be helpful for guiding treatment and improving patients’ survivals. The Surveillance, Epidemiology and End Results (SEER) database in the United States covers approximately 47.9% of the US population and collects data on patient demographic characteristics, primary tumor sites, tumor morphology, stage at diagnosis, first course of treatment, patients vital statuses, and morbidity data (5). The 2022 statistics from the the SEER database show 1980 new cases of ovarian cancer and a five-year relative survival rate of 49.7% for ovarian cancers diagnosed between 2012 and 2018 (6). EOC is the most common type of ovarian cancer, accounting for more than 90% of cases (7); and, according to the 2020 World Health Organization (WHO) histopathological classification of ovarian tumors, EOC includes plasmacytomas, mucinous tumors, endometrioid tumors, clear cell tumors, and other epithelial ovarian tumors (Brenner tumors, other types of cancer, and mesenchyma-derived tumors) (8).

The SEER database has been kept up-to-date since its inception, and it contains basic information such as clinical diagnoses, treatments and prognosis survivals, providing a large amount of reliable data for prognosis survival analyses. Clinical practice prognoses based on tumor stages alone are unreliable, but considering different variables together such as the tumor stage, age, histological type, differentiation, and treatment features may improve the accuracy of prognoses; we aimed to construct and validate a clinical prognostic model for ovarian cancer based on the data available in the SEER database.

## 2 Materials and methods

### 2.1 Patient selection

We conducted this study using data from elderly women with pathologically confirmed EOC entered in the US SEER database (version 8. 4.1) between 2010 and 2019. The data are publicly accessible without the need for an ethics committee review or approval, or patient informed consents. We selected on the basis of the following inclusion criteria: women older than 70 years, who met the 2020 WHO classification criteria for female genital tumors with pathologically diagnosed EOC (ICD-O-3 code C56. 9) and complete clinical information (including age, race, marital status, histological type, stage, and treatment plan). We excluded data from women with other malignancies, or unknown tumor-specific survivals or overall survivals (in months), or unknown tumor stages.

### 2.2 Outcome indicators

We selected the following 17 indicators as candidate prognosis predictors: age, race, marital status, histological type, AJC staging, cell differentiation degree, preoperative serum CA125 level, unilateral or bilateral tumor involvement, tumor size, extent of lymph node surgery, number of positive regional lymph nodes, size of residual lesions after tumor cytoreductive surgery, chemotherapy, initial site surgery information, sequence of systemic versus surgical treatment, organ metastasis (bone, brain, liver, lung), and time from diagnosis to treatment. We recorded the overall survival (OS) in months at the end of the follow-up time.

### 2.3 Analysis methods

After screening patient data for inclusion and exclusion criteria, we randomly grouped the relevant data from each patient collected from the SEER database using R Studio version 4.2.2 software, dividing them at a 7:3 ratio into a training and a validation sets. We analyzed the data from the two groups using chi-squared tests, with *P* < 0.5 representing statistically significant differences.

#### 2.3.1 Cox regression analysis to determine independent prognostic factors

We subjected candidate prognostic factors to one-way Cox regression analysis in the training set, and included the resulting statistically significant (*P* < 0.5) prognostic factors in the multi-factor Cox regression analysis to obtain the independent prognostic factors for EOC; in addition, we derived the risk ratio (HR) and 95% confidence interval (CI) of each candidate prognostic factor and used the least absolute shrinkage and selection operator (LASSO) to further identify the screened independent prognostic factors.

#### 2.3.2 Construction of survival nomogram

We included the independent prognostic factors obtained by regression analysis in the training set, and we drew a survival chart of the prognoses of elderly women with EOC. According to the different clinical characteristic groups, we entered the corresponding patients’ scores in the line chart and we added all the scores to get the total score to predict the 1-year, 3-year and 5-year OSs values for elderly women with EOC. We applied the consistency index (C-index) to evaluate the prediction accuracy of an event; we used the area under curve (AUC) to evaluate the differentiation of the line map, and the calibration curve to verify the consistency of the probability between the overall survival time and the actual survival time of the cancer; finally, we assessed the clinical applicability of the model line map obtained using a decision curve analysis (DCA).

## 3 Results

**Figure 1:**
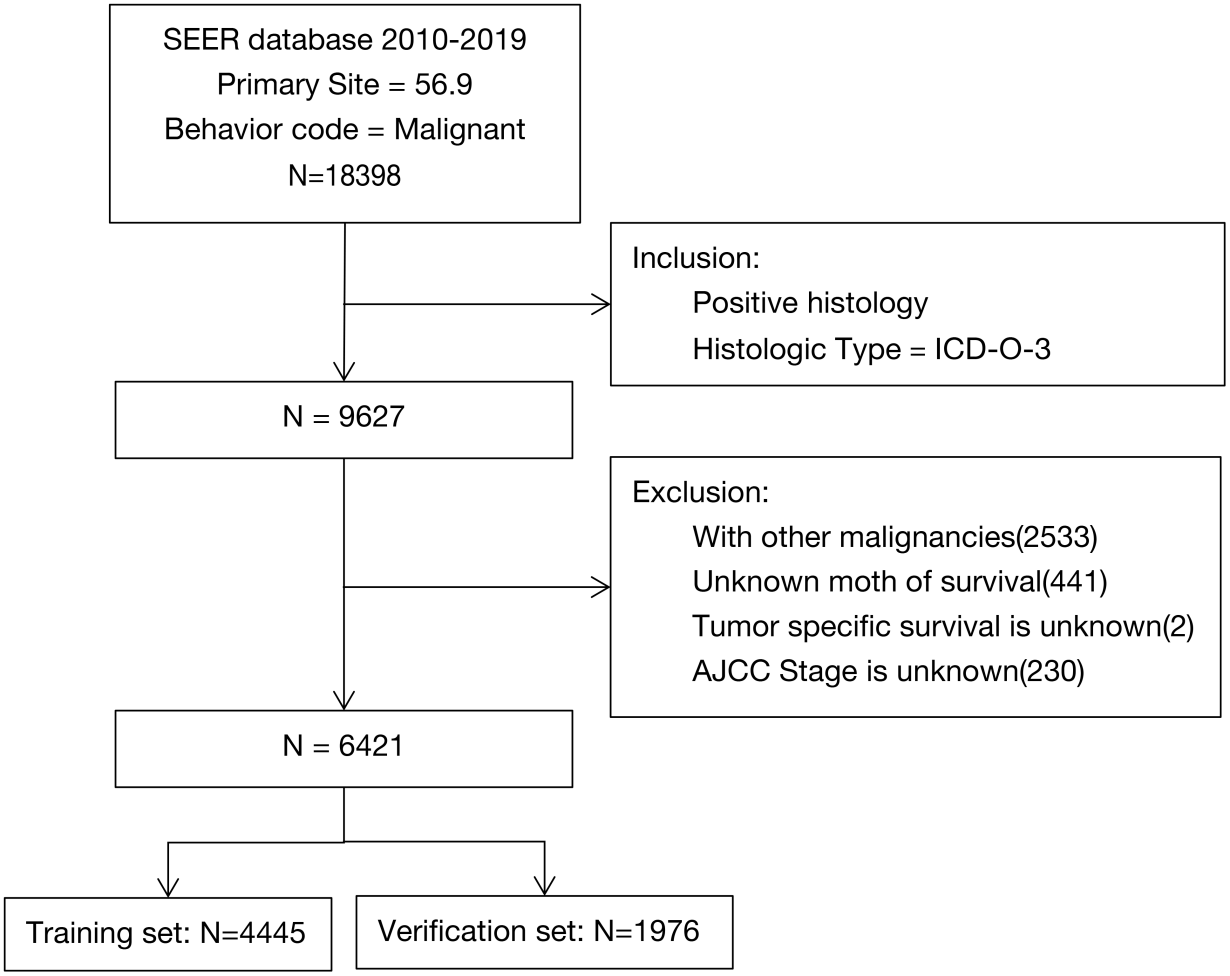
SEER screening process.

We included clinical data of 6421 elderly women with EOC from the SEER database in our analyses and randomized them into two groups (4445 cases in the training set and 1976 cases in the verification set; **Figure 1**). After chi-square tests, we found no significant differences between the two data sets (*P*>0.05, **Table 1**).

**Table 1:** Clinicopathological data of patients with EOC.

Variable	Total (%)	Training set (%)	Verification set (%)	*P*
Age (years)				0.529
70-74	2698 (42.0)	1890 (42.5)	808 (40.9)	
75-79	1855 (28.9)	1259 (28.3)	596 (30.2)	
80-85+	1868 (29.1)	1296 (29.2)	572 (28.9)	
Race				0.494
White	5538 (86.2)	3843 (86.4)	1695 (85.8)	
Black	402 (6.3)	274 (6.2)	128 (6.5)	
Others	481 (7.5)	328 (7.4)	153 (7.7)	
Marital Status				0.700
Single	641 (10.0)	442 (9.9)	199 (10.1)	
Married	2825 (44.0)	1941 (43.7)	884 (44.7)	
Widowed	1976 (30.8)	1390 (31.3)	586 (29.7)	
Other	979 (15.2)	672 (15.1)	307 (15.5)	
Histologic				0.759
Serous	5049 (78.6)	3491 (78.5)	1558 (78.8)	
Mucionous	277 (4.3)	192 (4.3)	85 (4.3)	
Endometrioid	528 (8.2)	365 (8.2)	163 (8.2)	
Clear cell	275 (4.3)	195 (4.4)	80 (4.1)	
Other epithelial	292 (4.6)	202 (4.6)	90 (4.6)	
AJCC				0.826
I	943 (14.7)	646 (14.5)	297 (15.0)	
II	525 (8.2)	362 (8.2)	163 (8.3)	
III	3010 (46.9)	2099 (47.2)	911 (46.1)	
IV	1943 (30.2)	1338 (30.1)	605 (30.6)	
Grade				0.776
Highly	665 (10.4)	460 (10.4)	205 (10.4)	
Moderate	454 (7.1)	325 (7.3)	129 (6.5)	
Poorly/ Undifferentiated	3198 (49.8)	2184 (49.1)	1014 (51.3)	
Blank	2104 (32.7)	1476 (33.2)	628 (31.8)	
CA125				0.772
Positive	4789 (74.6)	3330 (74.9)	1459 (73.8)	
Negative/Borderline	459 (7.1)	313 (7.0)	146 (7.4)	
Unknown/Blank	1173 (18.3)	802 (18.1)	371 (18.8)	
Laterality				0.706
Unilateral	3332 (51.9)	2303 (51.8)	1029 (52.1)	
Bilateral	2158 (33.6)	1491 (33.6)	667 (33.8)	
Paired	931 (14.5)	651 (14.6)	280 (14.2)	
Tumor size (cm)				0.665
Blank	2021 (31.5)	1393 (31.4)	628 (31.8)	
≤10	2795 (43.5)	1935 (43.5)	860 (43.5)	
>10	1605 (24.0)	1117 (25.1)	488 (24.7)	
Number of poditive LN				0.147
Negative	1629 (25.4)	1102 (24.8)	527 (26.7)	
≥1	1019(15.8)	711 (16.0)	308 (15.6)	
Unknown	3773 (58.8)	2632 (59.2)	1141 (57.7)	
Residual lesion (cm)				0.327
No/Blank	3668 (57.1)	2523 (56.8)	1145 (57.9)	
R0	1801 (28.0)	1260 (28.3)	541 (27.4)	
R1:≤1	587 (9.2)	397 (8.9)	190 (9.6)	
R2:>1	365 (5.7)	265 (6.0)	100 (5.1)	
Chemotherapy				0.196
No	1568 (24.4)	1106 (24.9)	462 (23.4)	
Yes	4853 (75.6)	3339 (75.1)	1514 (76.6)	
Surgery				0.288
Yes	5244 (81.7)	3615 (81.3)	1629 (82.4)	
No	1177 (18.3)	830 (18.7)	347 (17.6)	
LN sugery scope				0.0612
No	3789 (59.0)	2651 (59.6)	1138 (57.6)	
Sentinel LN biopsy	78 (1.2)	63 (1.4)	15 (0.8)	
1-3	644 (10.0)	434 (9.8)	210 (10.6)	
≥4	1807 (28.2)	1232 (22.7)	575 (29.1)	
Blank	103 (1.6)	65 (1.5)	38 (1.9)	
Systemic threapy/Surgery sequence				0.127
No	2178 (33.9)	1527 (34.4)	651 (32.9)	
Systemic treatment before surgery	589 (9.2)	419 (9.4)	170 (8.6)	
Systemic treatment intraoperative/after surgery	2754 (42.9)	1889 (42.5)	865 (43.8)	
Systemic treatment both before and after surgery	844 (13.1)	571 (12.8)	273 (13.8)	
Surgery both before and after systemic treatment	56 (0.9)	39 (0.9)	17 (0.9)	
Bone				0.590
No	6241 (97.2)	4316 (97.1)	1925 (97.4)	
Yes	45 (0.7)	34 (0.8)	11 (0.6)	
Unknown	135 (2.1)	95 (2.1)	40 (2.0)	
Brain				0.494
No	6276 (97.7)	4341 (97.7)	1935 (97.9)	
Yes	6 (0.1)	4 (0.1)	2 (0.1)	
Unknown	139 (2.2)	100 (2.2)	39 (2.0)	
Liver				0.593
No	5883 (91.6)	4068 (91.5)	1815 (91.9)	
Yes	399 (6.2)	278 (6.3)	121 (6.1)	
Unknown	139 (2.2)	99 (2.2)	40 (2.0)	
Lung				0.739
No	5965 (92.9)	4127 (96.0)	1838 (93.0)	
Yes	316 (4.9)	219 (2.9)	97 (4.9)	
Unknown	140 (2.2)	99 (1.1)	41 (2.1)	
Months from diagnosis to treatment				0.600
<1 month	3623 (56.4)	2518 (56.6)	1105 (55.9)	
1-3 months	2370 (36.9)	1616 (36.4)	754 (38.1)	
>3 months	63 (1.0)	48 (1.1)	15 (0.8)	
Blank	365 (5.7)	263 (5.9)	102 (5.2)	

### 3.1 Model results

**Figure 2:**
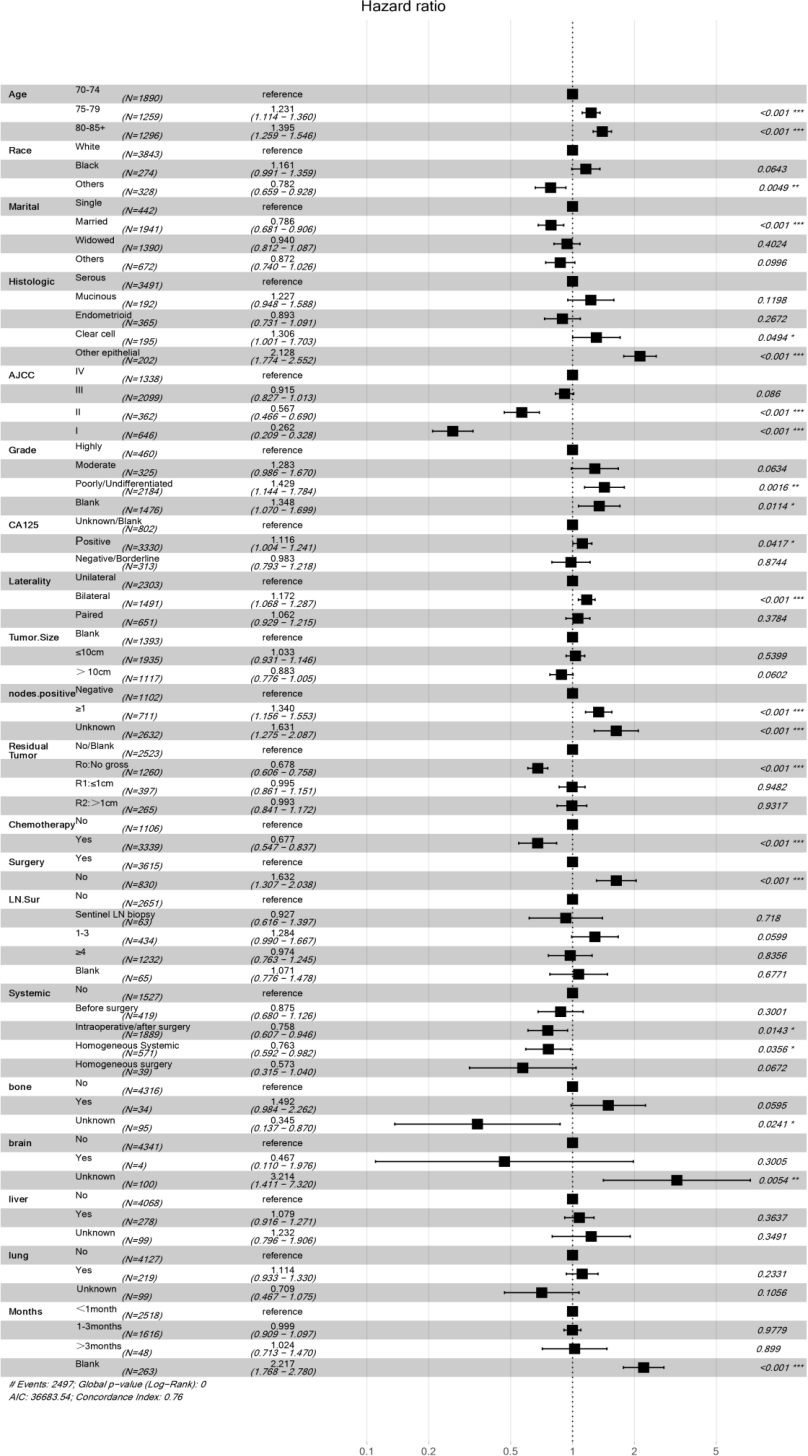
Multivariate regression model of women with EOC in training concentration. *P<0.05, **P<0.01, ***P<0.001, Statistically signicant difference, the significance increased successively. “#” It only stands for remarks.

**Figure 3:**
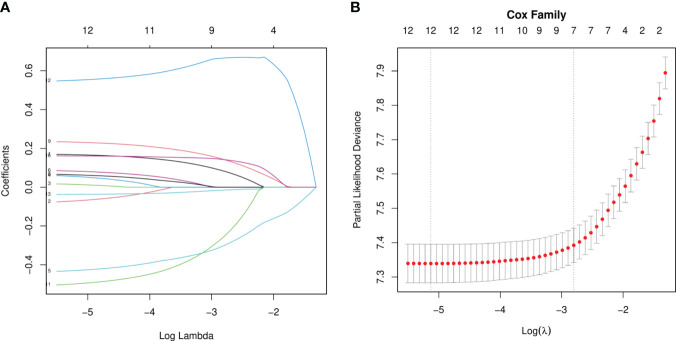
LASSO Regression analysis. **(A)** LASSO Coefficient distribution map-LASSO coefficient distribution of all variables. **(B)** variables determined by LASSO analysis (n=12).

**Figure 4:**
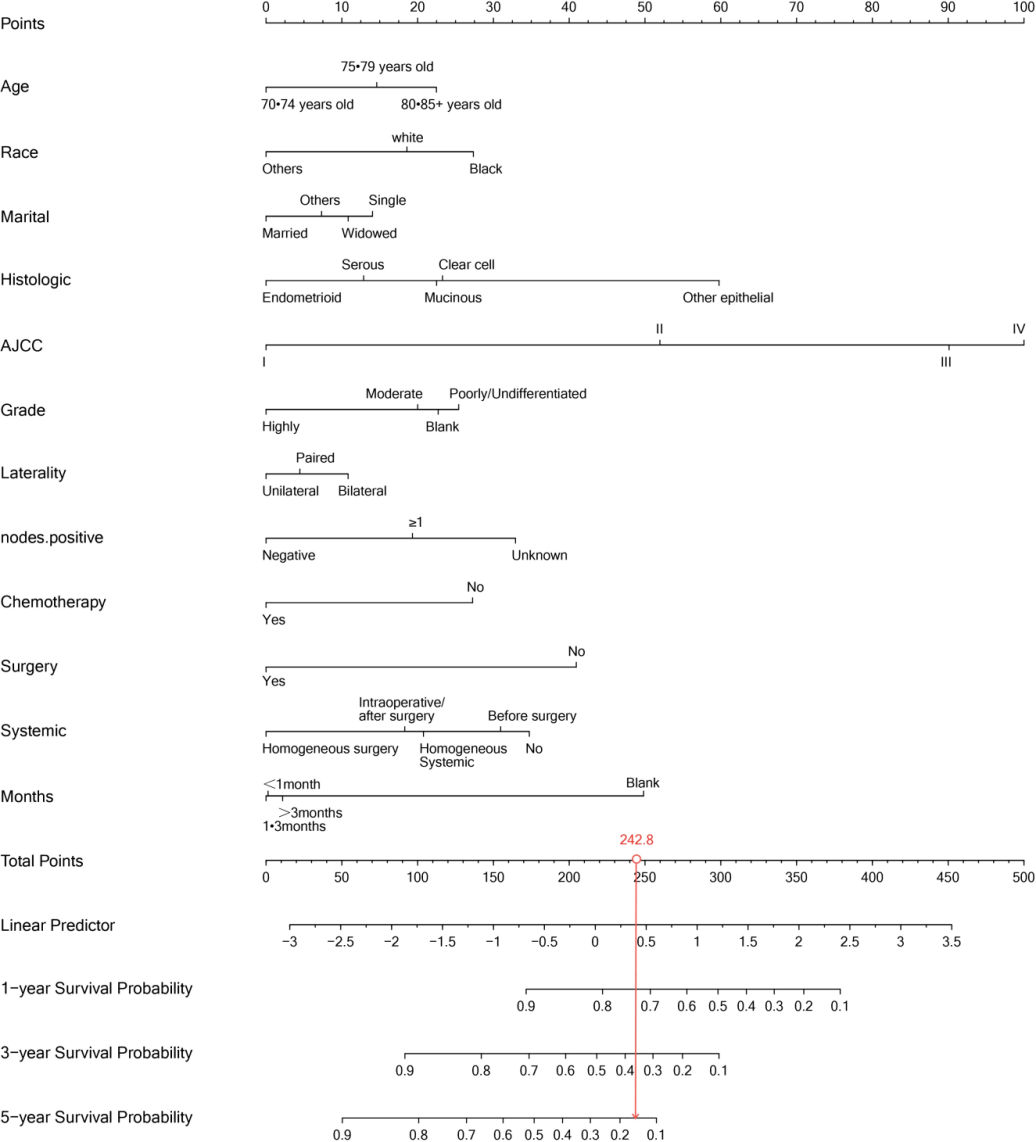
Line chart model of 1-year, 3-year and 5-year OS in elderly women with EOC.

Our univariate analysis of the candidate predictors in the training set showed that the 17 predictive factors selected had an impact on the prognosis and survival of elderly women with EOC (P < 0.05; **Table 2**). The results of our multivariate analysis and forest map (**Figure 2**) show that, except for the tumor size and the scope of lymph node operation, the candidate predictors were independent risk factors for elderly women with EOC. Moreover, after eliminating the three factors with a coefficient of 0 (residual lesion size, preoperative serum CA125 level, and organ metastasis after tumor cell reduction) and performing a LASSO regression, the remaining 12 influencing factors did not need to be further excluded (**Figure 3**). The survival chart shows that each independent prognostic factor corresponds to a score. By adding the scores of independent prognostic factors of a patient, a specific total score can be obtained, corresponding to the 1-year, 3-year and 5-year OS values for that patient. For example, a 78-year-old white women with EOC widowed at the age of 78, who had moderately differentiated serous bilateral tumors, AJCC stage III, more than one positive lymph node, surgical treatment, systemic treatment during or after the operation, a time from diagnosis to treatment within 1–3 months, but no postoperative chemotherapy had a total score of 242.8; and her corresponding 1-year survival rate was higher than 70%, her 3-year survival rate was less than 40%, and her 5-year survival rate was only approximately 15% (**Figure 4**).

**Table 2:** Univariate and multivariate Cox regression analyses of patients in training set.

Variable	Single factor analysis		Multi-factor analysis	
	HR 95%CI	*P*	HR 95%CI	*P*
Age (years)				
70-74	1.00		reference	
75-79	1.266 (1.148,1.396)	<0.001^***^	1.231 (1.114,1.360)	<0.001^***^
80-85+	1.841 (1.677,2.021)	<0.001^***^	1.395 (1.259,1.546)	<0.001^***^
Race				
White	1.00		reference	
Black	1.270 (1.087,1.482)	0.0025^**^	1.161 (0.991,1.359)	0.0643
Others	0.719 (0.607,0.851)	<0.001^***^	0.782 (0.659,0.928)	0.0049 ^**^
Marital				
Single	1.00		reference	
Married	0.802 (0.697,0.922)	0.0020^**^	0.786 (0.681,0.906)	<0.001^***^
Widowed	1.154 (1.002,1.330)	0.0471^*^	0.940 (0.812,1.087)	0.4024
Others	0.971 (0.826,1.140)	0.7184	0.872 (0.740,1.026)	0.0996
Histologic				
Serous	1.00		reference	
Mucionous	0.517 (0.409,0.652)	<0.001^***^	1.227 (0.948,1.588)	0.1198
Endometrioid	0.387 (0.323,0.465)	<0.001^***^	0.893 (0.731,1.091)	0.2672
Clear cell	0.428 (0.334,0.548)	<0.001^***^	1.306 (1.001,1.703)	0.0494^*^
Other epithelial	1.433 (1.204,1.706)	<0.001^***^	2.128 (1.774,2.552)	<0.001^***^
AJCC				
IV	1.00		reference	
III	0.668 (0.613,0.727)	<0.001^***^	0.915 (0.827,1.013)	0.0860
II	0.359 (0.301,0.427)	<0.001^***^	0.567 (0.466,0.690)	<0.001^***^
I	0.171 (0.143,0.203)	<0.001^***^	0.262 (0.209,0.328)	<0.001^***^
Grade				
Highly	1.00		reference	
Moderate	1.332 (1.026,1.730)	0.0313^*^	1.283 (0.986,1.670)	0.0634
Poorly/ Undifferentiated	2.267 (1.837,2.797)	<0.001^***^	1.429 (1.144,1.784)	0.0016^**^
Blank	3.365 (2.717,4.167)	<0.001^***^	1.348 (1.070,1.699)	0.0114^*^
CA125				
Unknown/Blank	1.00		reference	
Positive	0.993 (0.899,1.098)	0.896	1.116 (1.004,1.241)	0.0417^*^
Negative/Borderline	0.486 (0.396,0.598)	<0.001^***^	0.983 (0.793,1.218)	0.8744
Laterality				
Unilateral	1.00		reference	
Bilateral	1.416 (1.297,1.547)	<0.001^***^	1.172 (1.068,1.287)	<0.001^***^
Paired	2.809 (2.523,3.127)	<0.001^***^	1.062 (0.929,1.215)	0.3784
Tumor size (cm)				
Blank	1.00		reference	
≤10	0.614 (0.563,0.670)	<0.001^***^	1.033 (0.931,1.146)	0.5399
>10	0.453 (0.405,0.505)	<0.001^***^	0.883 (0.776,1.005)	0.0602
Number of positive LN				
Negative	1.00		reference	
≥1	2.128 (1.854,2.442)	<0.001^***^	1.340 (1.156,1.553)	<0.001^***^
Unknown	2.825 (2.527,3.158)	<0.001^***^	1.631 (1.275,2.087)	<0.001^***^
Residual lesion (cm)				
No/Blank	1.00		reference	
R0	0.405 (0.367,0.448)	<0.001^***^	0.678 (0.606,0.758)	<0.001^*^
R1:≤1	0.803 (0.702,0.918)	0.0014^**^	0.995 (0.861,1.151)	0.9482
R2:>1	0.900 (0.769,1.053)	0.1868	0.993 (0.841,1.172)	0.9317
Chemotherapy				
No	1.00		reference	
Yes	0.694 (0.636,0.757)	<0.001^***^	0.677 (0.547,0.837)	<0.001^***^
Surgery				
Yes	1.00		reference	
No	4.157 (3.797,4.552)	<0.001^***^	1.632 (1.307,2.038)	<0.001^***^
LN surgery scope				
No	1.00		reference	
Sentinel LN biopsy	0.789 (0.562,1.108)	0.1710	0.927 (0.616,1.397)	0.7180
1-3	0.669 (0.583,0.767)	<0.001^***^	1.284 (0.990,1.667)	0.0599
≥4	0.420 (0.380,0.464)	<0.001^***^	0.974 (0.763,1.245)	0.8356
Blank	0.871 (0.650,0.168)	0.3570	1.071 (0.766,1.478)	0.6771
Systemic treatment/Surgery sequence				
No	1.00		reference	
Systemic treatment before surgery	0.730 (0.637,0.835)	<0.001^***^	0.875 (0.680,1.126)	0.3001
Systemic treatment intraoperative/after surgery	0.455 (0.417,0.498)	<0.001^***^	0.758 (0.607,0.982)	0.0143^*^
Systemic treatment both before and after surgery	0.600 (0.525,0.685)	<0.001^***^	0.763 (0.592,0.982)	0.0356^*^
Surgery both before and after systemic treatment	0.388 (0.224,0.670)	<0.001^***^	0.573 (0.315,1.040)	0.0672
Bone				
No	1.00		reference	
Yes	2.450 (1.651,3.634)	<0.001^***^	1.492 (0.984,2.262)	0.0595
Unknown	2.252 (1.802,2.814)	<0.001^***^	0.345 (0.137,0.870)	0.0241^*^
Brain				
No	1.00		reference	
Yes	0.987 (0.247,3.951)	0.9860	0.467 (0.110,1.976)	0.3005
Unknown	2.364 (1.904,2.935)	<0.001^***^	3.214 (1.411,7.320)	0.0054^**^
Liver				
No	1.00		reference	
Yes	1.767 (1.525,2.048)	<0.001^***^	1.079 (0.916,1.271)	0.3637
Unknown	2.255 (1.809,2.812)	<0.001^***^	1.232 (0.796,1.906)	0.3491
Lung				
No	1.00		reference	
Yes	1.865 (1.585,2.194)	<0.001^***^	1.114 (0.933,1.330)	0.2331
Unknown	2.085 (1.673,2.599)	<0.001^***^	0.709 (0.467,1.075)	0.1056
Months from diagnosis to treatment				
<1 month	1.00		reference	
1-3 months	1.440 (1.323,1.568)	<0.001^***^	0.999 (0.909,1.097)	0.9779
>3 months	1.790 (1.253,2.556)	0.0014	1.024 (0.713,1.470)	0.8990
Blank	8.579 (7.448,9.883)	<0.001^***^	2.217 (1.768,2.780)	<0.001^***^

*P<0.05, **P<0.01, ***P<0.001, Statistically signicant difference, the significance increased successively.

### 3.2 Verification results

**Figure 5:**
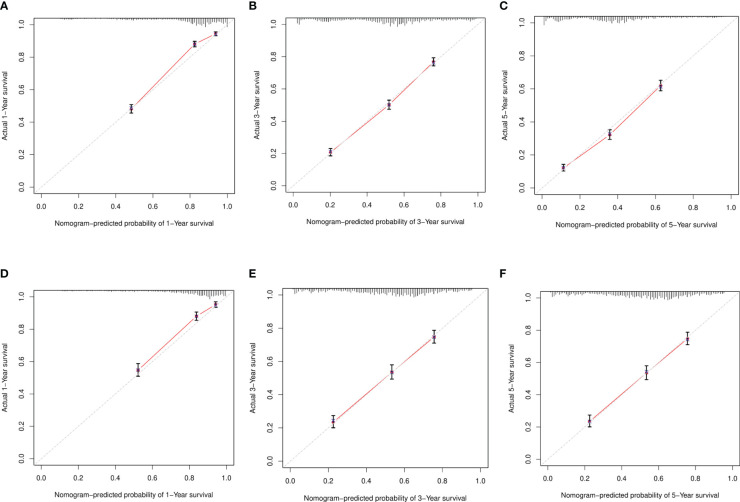
Calibration curve. **(A–C)** Training sets at 1 year, 3 years, and 5 years. **(D–F)** Validation sets at 1 year, 3 years, and 5 years.

To verify the accuracy of the model, we drew a calibration curve with a slope of 1 as a reference between the training and verification sets. The calibration curve we obtained exhibited a close resemblance to the ideal curve, proving the adequacy of our line chart model and the consistency of the predicted survival rates with the actual survival rates observed (**Figure 5**).

**Figure 6:**
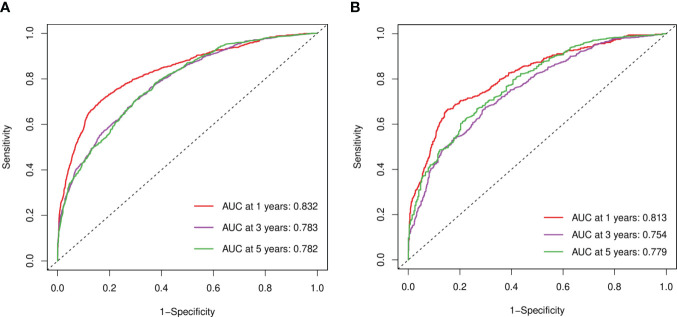
ROC curves. **(A)** Training sets at 1, 3, and 5 years. **(B)** Validation sets at 1, 3, and 5 years.

In the training and verification sets, the C indexes of elderly women EOC were 0.749 and 0.735 respectively, indicating that the prediction accuracy of the line chart was high. According to the ROC curve in the training set, the 1-year AUC was 0.832, the 3-year AUC was 0.783, and the 5-year AUC was 0.782, and in the verification set the 1-year AUC was 0.813, the 3-year AUC was 0.754, and the 5-year AUC was 0.779. The AUCs of both sets were higher than 0.7 and the results were similar, indicating that the prediction model discrimination is high (**Figure 6**).

### 3.3 Risk stratification and decision curve

**Figure 7:**
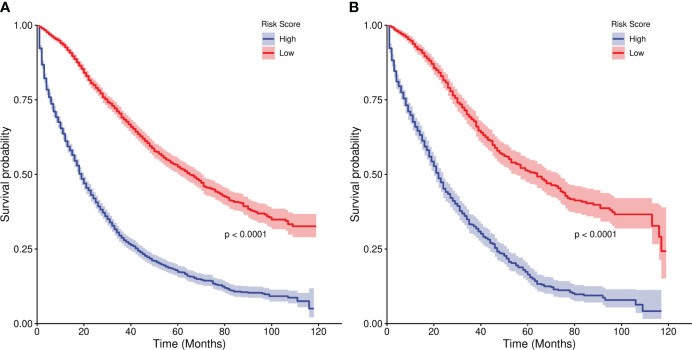
Kaplan-Meier curves of risk stratification. **(A)** Training set. **(B)** validation set.

We divided the risk scores of independent prognostic factors into low risk and high risk groups according to the median risk scores of the independent prognostic factors. In the training set,the median survival times of the high risk group were 19 months, and the low risk group were 65 months. In the Verification set, the median survival times of the high risk group were 22 months, and the low risk group were 64 months. The Kaplan-Meier curves in **Figure 7** show that the 1-, 3- and 5-year survival prognoses of the low-risk group were significantly better than those of the high-risk group (P < 0.001), demonstrating the robust risk stratification ability of our line chart.

**Figure 8:**
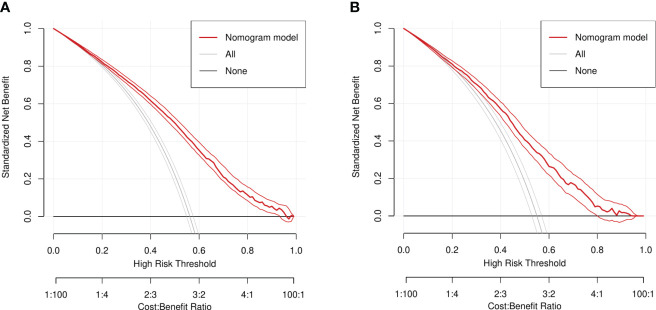
DCA curves. **(A)** Training set. **(B)** validation set.

In addition, combined with the results of the DCA curve(Generally, the farther the model curve is from all or none curves, the higher the clinical application value of the model curve). It can be clearly concluded from the results that in the DCA curves of the training set and the validation set, the threshold of patients who can benefit is basically aroud 20%-95%. Therefore, the line chart model can be used to effectively predict the 1-, 3- and 5-year overall survival rates of elderly women with EOC (**Figure 8**).

## 4 Discussion

In this study, we analyzed multiple independent factors for EOC in elderly women, and our results show that the survival rates predicted by our model line chart align well with the actual survival rates, indicating that the clinical prognostic model is useful to evaluate the survival prognosis of elderly women with EOC.

Ovarian cancer may occur in any age group, but it is more common in patients older than 50 years (2). We included data from patients with EOC older than 70 years in this study. Our line chart predicts a worsening prognosis with age increases. We believe that age is a risk factor for ovarian cancer with older women being at higher risk of developing invasive tumors. In addition, the physical function decline of older women results in relatively poor survival prognoses, a finding consistent with those of Pawelec G et al. (9).

Zeng C et al. found that the ethnic survival gap is widening in patients with ovarian cancer (10). Factors such as poverty and lack of access to health care may affect the outcome of diseases like ovarian cancer (11). On the basis of the results of the African American Cancer Epidemiological study (AACES), Schildkraut JM et al. compared cases in the SEER database and found that black women with lower levels of income (45% < $25000 per year), education (51% ≤ high school education), and insurance coverage (32% without insurance or Medicaid) had poorer prognoses than white women in the SEER database (12). This suggests that ethnic differences in the survival rate of ovarian cancer are associated with the stage of diagnosis, the quality of care, and social factors of health (11, 13). More than 90% of the data in this study are derived from Caucasian and black women, and most are derived from Caucasian women. The prognosis differences may be related to a lack of supportive care for black women.

Our results show that the OS values of unmarried elderly women with EOC are significantly lower than those of married or widowed elderly women. This is consistent with the results of other studies (14): compared with married elderly women with EOC, unmarried women with EOC have a higher risk of late diagnosis and worse survival outcomes. The reason for this may be related to a higher number of ovulation cycles in unmarried women, who are also more likely to develop malignant tumors. The risk of ovarian cancer increases in women with more ovulation cycles, such as those with younger menarche and later menopause (15, 16). Therefore, unmarried elderly women with ovarian cancer require special attention during their diagnoses and treatments.

Tumoral histological types and cell differentiation degree have an important role in the prognosis and survival of elderly women with EOC. EOC is the most common ovarian cancer, and high-grade serous ovarian cancer is its most common type and one of the most aggressive histological types, accounting for about 70% of newly diagnosed cases (17). Endometrioid tumors, clear cell tumors, and other types of tumors account for the remaining 30%. The prognosis of serous tumors is better than those of mucinous, clear cell, and other epithelial ovarian cancers. Usually, EOC is diagnosed by histopathological evaluation after ovariectomy, or fallopian tube or peritoneal biopsy; and, the treatment regimen is individualized according to the histopathological type, stage, and individual symptoms of each patient (18). However, studies have found that (19) only approximately 13% of serous ovarian cancer cases are diagnosed during stages I or II; the 10-year survival rate for early diagnoses is 55% and that for late diagnoses reaches only 15%. Moreover, most of these patients are diagnosed because they develop symptoms of distant metastasis, which have a great impact on the prognoses of the patients. Other studies (20, 21) have shown that women with serous ovarian cancers have a worse prognosis than those with mucinous or clear cell cancers. Howere, in our study, the prognoses of serous ovarian cancer have been found to be significantly better than that of ovarian mucinous and clear cell tumors, and when compared with other EOC histological types (regardless of stage and differentiation degree), the prognoses of endometrioid carcinomas have been the best (22); we found results consistent with the evidence. These findings may be associated with epigenetic mechanisms of ovarian cancer (23), the unique clinical characteristics of each tumor subtype, and different gene mutation sites (24). Our nomogram model also confirmed that worse degrees of cell differentiation result in lower 1-, 3- and 5-year overall survival rates.

Ovarian cancer (especially advanced ovarian cancer) remains one of the gynecological tumors with most unfavorable prognoses (25). For this study, we used the staging system of the American Joint Commission on Cancer (AJCC), which includes the traditional TNM staging and the more commonly used International Federation of Obstetrics and Gynecology staging (26). Because early ovarian cancer is usually asymptomatic and difficult to detect, most patients are diagnosed during an advanced stage. We found that more than 70% of the patients were diagnosed with advanced stages, and our results showed that advanced tumor stages led to the worse survival prognoses. This is because the lesions can be more easily removed in patients with early tumor stages, and residual lesions are relatively small and sensitive to chemotherapy, resulting in low risks of recurrence and metastases. According to AJCC staging, patients with lymph node metastasis have stages III or higher, and patients with liver parenchyma metastasis have stage IV cancer. The prognosis differences between early stage patients and late stage patients were more pronounced after the multivariate analysis (*P* < 0.001). Our line chart shows that the assigning scores of women with late stage EOC (III stage, 90.38; IV stage, 100) were significantly higher than those of early stage patients (stage I, 0; stage II, 53.31). Li X et al. (27) also have suggested that early detection, early diagnosis, and early treatment are necessary to improve the long-term prognoses of patients with EOC. This confirms that the survival rates of patients with lymph node or liver parenchyma metastases are lower than those of patients without metastasis.

According to the results of a primary debulking surgery, the surgical satisfaction can be classified into three categories (28): complete removal of tumor or visual absence of residual tumor (R0), maximum diameter of single residual lesion ≤ 1 cm (R1), maximum diameter of single residual tumor >1 cm (R2). In this study, univariate Cox regression showed that R0 was an influencing factor for EOC prognoses in elderly women, but urther screening by LASSO regression analysis showed that the size of residual lesions after tumor cell reduction was an independent risk factor for the prognosis. According to the latest NCCN Guide(1^st^ edition, 2023) (29), full staging during surgery has not been demonstrated to improve the survival of patients with R0, but it is important to determine the most appropriate postoperative treatment. Our results support this notion.

The latest guidelines for the use of the SEER database state clearly that chemotherapy, hormone therapy, biological response therapy/immunotherapy, and surgical/radiation endocrine therapy are considered systemic therapies (30). Our results show that postoperative chemotherapy is an independent prognostic factor for elderly women with EOC (*P* < 0.001). The overall survivals of patients after chemotherapy are significantly higher than those of patients without chemotherapy. For systemic chemotherapy, adjuvant therapy with immune checkpoint inhibitors (29) should be given after tumor cell reduction in women with stages II-IV EOC, and platinum-based chemotherapy is recommended. The recommendation includes six courses of standard paclitaxel (paclitaxel or docetaxel) + platinum (carboplatin or cisplatin) intravenous chemotherapy (29, 31). Our multivariate analysis results show that systemic treatment and surgical operation were independent prognostic factors in elderly women with EOC, and their prognoses were significantly higher than those of women without systemic treatment. However, our results showed similar prognoses after either postoperative or intraoperative systemic treatment (systemic treatment options are performed during surgery, for example, intraperitoneal perfusion chemotherapy or radiotherapy, etc). Although new radiotherapy techniques have been gradually applied in recent years and are relatively effective, radiotherapy is still not the preferred adjuvant therapy in ovarian cancer (32).

The serum levels of CA125 (a marker of EOC) may increase in women with ovarian cancer, but the sensitivity of this marker is low during the early stages (33). High preoperative CA125 levels have been associated with worse EOC prognoses (34, 35). In our study with data from elderly women, the univariate analysis showed a significant difference between patients with elevated serum CA125 and patients with normal preoperative CA125 levels, but the multivariate analysis failed to identify serum CA125 as an independent prognostic factor, this finding differs from those in other studies. However, the levels of CA125 in the study by Dikmen et al. (36) suggest that the marker is less than ideal for the diagnosis of ovarian cancer.

Studies have not found an association between the tumor diameter and the prognosis of EOC. In fact, a number of scholars (37, 38) have shown that the tumor diameter in patients with early EOC is larger than that in patients with advanced stages. In our study, of the 4953 women with advanced III-IV stages (77. 1%), only 984 cases (19.9%) had tumor diameters larger than 10 cm, and more than 2182 cases (40%) had tumor diameters smaller than or equal to 10 cm. Thus, most patients with advanced disease had relatively small tumor diameters. We divided tumor diameter data into two groups with 10 cm as the dividing point. The univariate analysis showed a significant difference between the prognoses of patients with tumors larger than 10 cm and those with tumors smaller than or equal to 10 cm (*P* < 0.001), but the multivariate analysis did not suggest that the tumor size was an independent factor affecting the prognosis of EOC. This may be due to the lack of a specific analysis of the different stages and tumor diameters in this study, and it may also be related to the fact that early EOC grows *in situ* without distant metastases, whereas advanced EOC tumors may be relatively small, but are often accompanied by metastases. Some researchers have indicated that the tumor size cannot be used to prognosticate EOC outcomes (39). It was considered that it might be related to the critical value selected by the grouping, and many different groups could be analyzed and compared later.

We are aware of the limitations of this study: 1) This was a retrospective study with clinical data from the SEER database on middle-aged and elderly women with EOC that may reflect selective biases. 2) We focused on women in the United States with data included in the SEER database, most of them were white, and we lack external clinical data to verify the accuracy of the entries. 3) A lot of basic clinical data are missing from this database, such as the presence of tumor markers (HE4, CA199, and CEA, *etc.*), ascites, gene detection, specific chemotherapy and systemic therapy details (drugs, doses, *etc.*), and intraoperative bleeding records.

## 5 Conclusions

To sum up, we generated a survival nomogram based on data from the SEER database including age, race, marital status, histological type, AJCC stage, differentiation degree, unilateral and bilateral tumor involvement, number of positive lymph nodes, sequence of chemotherapy, surgery, systemic treatment and operation, and diagnosis to treatment time. The clinical prediction model is accurate for women older than 70 years with EOC, and it can provide a clinical basis for individualized treatment after operation.

## Data availability statement

Publicly available datasets were analyzed in this study. This data can be found here: Surveillance, Epidemiology and End Results (SEER) database.

## Ethics statement

Ethical approval was not required for the studies involving humans because We conducted this study using data from elderly women with pathologically confirmed EOC entered in the US SEER database between 2010 and 2019. The data are publicly accessible without the need for an ethics committee review or approval, or patient informed consents. The studies were conducted in accordance with the local legislation and institutional requirements. Written informed consent for participation was not required from the participants or the participants’ legal guardians/next of kin in accordance with the national legislation and institutional requirements because We conducted this study using data from elderly women with pathologically confirmed EOC entered in the US SEER database between 2010 and 2019. The data are publicly accessible without the need for an ethics committee review or approval, or patient informed consents. Written informed consent was not obtained from the individual(s) for the publication of any potentially identifiable images or data included in this article because We conducted this study using data from elderly women with pathologically confirmed EOC entered in the US SEER database between 2010 and 2019. The data are publicly accessible without the need for an ethics committee review or approval, or patient informed consents.

## Author contributions

YZ: Conceptualization, Data curation, Formal Analysis, Methodology, Project administration, Supervision, Visualization, Writing – original draft, Writing – review & editing. AW: Data curation, Formal Analysis, Project administration, Writing – review & editing. XS: Data curation, Formal Analysis, Writing – review & editing. RZ: Data curation, Writing – review & editing. LZ: Conceptualization, Formal Analysis, Funding acquisition, Methodology, Project administration, Supervision, Writing – original draft, Writing – review & editing.
